# Energy Landscapes in Hydrothermal Chimneys Shape Distributions of Primary Producers

**DOI:** 10.3389/fmicb.2018.01570

**Published:** 2018-07-16

**Authors:** Håkon Dahle, Sven Le Moine Bauer, Tamara Baumberger, Runar Stokke, Rolf B. Pedersen, Ingunn H. Thorseth, Ida H. Steen

**Affiliations:** ^1^K.G. Jebsen Centre for Deep Sea Research, University of Bergen, Bergen, Norway; ^2^Department of Biology, University of Bergen, Bergen, Norway; ^3^Pacific Marine Environmental Laboratory (NOAA), Newport, OR, United States; ^4^Department of Earth Science, University of Bergen, Bergen, Norway

**Keywords:** geobiology, geochemistry, microbiology, thermodynamics, modeling, ecology, hydrothermal systems

## Abstract

Hydrothermal systems are excellent natural laboratories for the study of how chemical energy landscapes shape microbial communities. Yet, only a few attempts have been made to quantify relationships between energy availability and microbial community structure in these systems. Here, we have investigated how microbial communities and chemical energy availabilities vary along cross-sections of two hydrothermal chimneys from the Soria Moria Vent Field and the Bruse Vent Field. Both vent fields are located on the Arctic Mid-Ocean Ridge, north of the Jan Mayen Island and the investigated chimneys were venting fluids with markedly different H_2_S:CH_4_ ratios. Energy landscapes were inferred from a stepwise *in silico* mixing of hydrothermal fluids (HFs) with seawater, where Gibbs energies of relevant redox-reactions were calculated at each step. These calculations formed the basis for simulations of relative abundances of primary producers in microbial communities. The simulations were compared with an analysis of 24 samples from chimney wall transects by sequencing of 16S rRNA gene amplicons using 454 sequencing. Patterns in relative abundances of sulfide oxidizing Epsilonproteobacteria and methane oxidizing Methylococcales and ANME-1, were consistent with simulations. However, even though H_2_ was present in HFs from both chimneys, the observed abundances of putative hydrogen oxidizing anaerobic sulfate reducers (Archaeoglobales) and methanogens (Methanococcales) in the inner parts of the Soria Moria Chimney were considerably higher than predicted by simulations. This indicates biogenic production of H_2_ in the chimney wall by fermentation, and suggests that biological activity inside the chimneys may modulate energy landscapes significantly. Our results are consistent with the notion that energy landscapes largely shape the distribution of primary producers in hydrothermal systems. Our study demonstrates how a combination of modeling and field observations can be useful in deciphering connections between chemical energy landscapes and metabolic networks within microbial communities.

## Introduction

All living organisms require a continuous supply of energy to sustain vital processes of nutrient uptake, growth, and repair. Hence, there must be a fundamental connection between energy availability and microbial community structure in terms of functional groups of organisms. Variations in concentrations of chemical species in the environment correspond to variations in the energy densities associated with specific chemical reactions. Hence, by determining the chemical composition and calculating Gibbs energies of relevant energy-yielding reactions in an environment, we can infer chemical energy-landscapes (i.e., distribution and magnitude of different chemical energy-sources), which can be quantitatively compared to observed microbial community structures (Dahle et al., 2015). Deep sea hydrothermal systems are well suited as natural laboratories for such studies (Dahle et al., 2015). Here, active hydrothermal chimneys are formed by mineral precipitation as hot, reduced and metal enriched fluids are emitted at the seafloor. The chimney walls are permeable, allowing gradual fluid mixing by an ingress of ambient seawater (SW) into the chimney interior and outpouring of hydrothermal fluids (HFs) (Goldfarb et al., 1983; Haymon, 1983; Kelley et al., 2002). This gives rise to chemical disequilibria supporting microbial communities driven by primary producers, oxidizing reduced chemical species from the high temperature fluids (e.g., H_2_, H_2_S, CH_4_), with electron acceptors from SW (e.g., O_2_, ${NO}_{3}^{-}$, ${SO}_{4}^{2-}$) (Baross and Hoffman, 1985; Jannasch and Mottl, 1985; Tivey, 1995). In addition, CO_2_ in the HFs may act as an electron acceptor for methanogens. Primary production may in turn support communities of organotrophs (Jannasch, 1995; Karl, 1995; Reysenbach et al., 2002; Miroshnichenko and Bonch-Osmolovskaya, 2006; Page et al., 2008).

The microbiology of hydrothermal chimneys from around the world has been explored in numerous studies involving cultivation (Gotz et al., 2002; Miroshnichenko and Bonch-Osmolovskaya, 2006; Reysenbach et al., 2006; Postec et al., 2007), 16S rRNA gene sequence profiling (e.g., Perner et al., 2007; Page et al., 2008; Flores et al., 2011, 2012; Dahle et al., 2015; Lin et al., 2016), and metagenomics and metatranscriptomics (Xie et al., 2011; Dahle et al., 2013; Stokke et al., 2015). These studies have revealed a remarkable phylogenetic and functional diversity. Chimney surfaces are typically colonized by aerobes such as sulfide and hydrogen oxidizing Epsilonproteobacteria and Aquificales, sulfide oxidizing Thiotrichales, and aerobic methane oxidizing Methylococcales, whereas anaerobes such as methane oxidizers of the ANME-clade, methanogenic Methanococcales, sulfate reducers (SRBs) such as Archaeoglobales, and organotrophs, such as Thermococcales and Thermotogales predominate in deeper layers. Yet, the underlying reasons for large variations between hydrothermal fields in relative abundances of organisms within these taxonomic and functional groups remain elusive.

Geochemical modeling combined with thermodynamic calculations suggest that energy landscapes vary considerably between vent fields as a consequence of variations in their geological setting (Tivey, 1995; Shock and Holland, 2004; McCollom, 2007; Amend et al., 2011; LaRowe et al., 2014; Dahle et al., 2015; Lin et al., 2016). For example, in basalt hosted systems, potential energy is typically mainly available as sulfide oxidation whereas in sediment influenced systems, considerable energy is also available from methane oxidation, hydrogen oxidation, and ammonium oxidation. However, quantitative comparisons between modeled energy landscapes and observed microbial communities in hydrothermal chimneys have been performed only recently (Dahle et al., 2015; Lin et al., 2016). These studies suggest that energy landscapes largely shape microbial communities, but also point at some discrepancies between models and observations, which may be ascribed to various factors such as contamination, uncertainties in functional assignments, as well as water-rock interactions or biotic and abiotic reactions not taken into account in the models. To further decipher the connections between energy availability and microbial communities in hydrothermal chimneys it is therefore important to obtain more data from systematic comparisons between models and observations from a wide range of samples associated with different geochemical settings.

Here we compare energy-based community structure modelling with analyses of microbial communities in hydrothermal chimney-walls from two hydrothermal fields. Unlike similar previous studies (Dahle et al., 2015; Lin et al., 2016), complete cross sections at down to millimeter scale resolution were analyzed, providing detailed information on how communities change from oxic to anaxic conditions.

Two chimneys were analyzed, one from the Soria Moria Vent Field, venting fluids with high H_2_S:CH_4_ ratios, and one from the recently discovered Bruse Vent Field, venting fluids with low H_2_S:CH_4_ ratios. The aim of the study was to investigate to what extent inferred relative energy densities arising from mixing between high temperature HFs and cold SW can explain observed variations in the distribution of functional groups of organisms within hydrothermal chimney walls. Our results provide evidence that energy landscapes largely shape the distribution of primary producers in these settings, particularly in outer surface layers and high-temperature inner layers. In intermediate parts of the chimney wall, complex metabolic food-webs seem to develop, arguably involving inter-species transfer of H_2_, making comparisons between models and observations more difficult to interpret.

## Materials and Methods

### Site Description, Sampling, and Chemical Analyses

**FIGURE 1:**
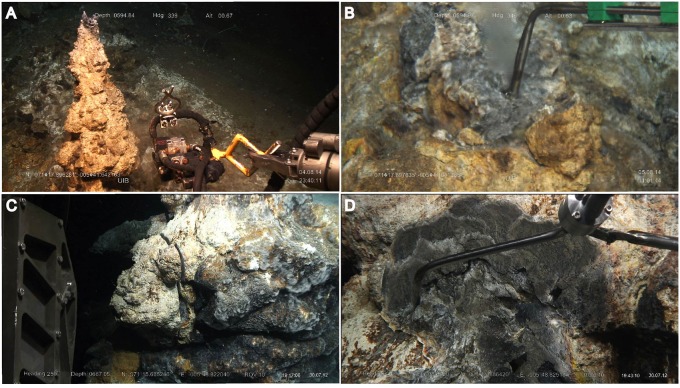
Sampling of chimneys and hydrothermal fluids. **(A)** The Bruse chimney before sampling. **(B)** Collection of hydrothermal fluids emitting through the central flow channel of the Bruse chimney after cutting with a chainsaw. **(C)** Sampling of the Soria Moria chimney using a chainsaw. **(D)** Collection of hydrothermal fluids emitted through a fracture in the cross section created during sampling of the Soria Moria chimney fragment.

The Arctic Mid-Ocean Ridge (AMOR), defining the Mid-Atlantic Ridge segments north of the Arctic Circle, is one of the most slow-spreading ridge systems on earth. The Soria Moria Vent Field (71°15′N, 05°49′W), located at a depth of around 700 m, was one of the first vent fields to be discovered at AMOR in 2005 (Pederesen et al., 2010). The Bruse Vent Field was discovered more recently (in 2014) about 5.5 km North-East from the Soria Moria Vent Field (71°18′N, 05°42′W) and at a depth of around 580 m. The samples used in this study were collected in July 2012 (Soria Moria chimney) and July 2014 (Bruse chimney) using a remotely operated underwater vehicle (ROV) during cruises with the G.O. Sars research vessel. The Bruse chimney was about 2 m high and emitted high temperature fluids (270°C) with no visible bubbles. Using a chainsaw, the chimney was cut close to its base, and water emitting from the central flow channel was collected (**Figures 1A,B**). The Soria Moria chimney was around 3 m tall, but did not emit a central flow of HFs from the top of the chimney. Instead, HFs were emitted through the chimney wall in multiple locations. A flange-like segment of the chimney was cut loose using a chainsaw (**Figure 1C**). This uncovered a fracture going from the center of the chimney into the sampled fragment. HFs emitting from the fracture were sampled directly after collection of the chimney fragment (**Figure 1D**).

Dissolved gasses were collected using titanium alloy gas tight bottles connected to a snorkel inlet operated by the ROV. To determine dissolved gas compositions (CO_2_, CH_4_, and H_2_) of the vent fluids, after each dive, the gas tight samples were processed on a seagoing high vacuum line to extract the gas phase (Lupton et al., 2006). Aliquots of the quantified gas were packaged in pyrex glass ampoules for later on-shore analysis at the University of Washington in Seattle, United States. Compositional analysis of the gas samples was accomplished using gas chromatography. Components were separated using either Hayesep A or Hayesep Q porous polymer columns started at 50°C and ramped to 120°C. Components were detected and quantified with FID and TCD detectors. SE for CO_2_, CH_4_, and H_2_ was ±3–5% of the measured value. To determine the non-volatile species of the vent fluids, samples were collected in 1000 ml titanium syringe samplers. Hydrogen sulfide (H_2_S), ${NH}_{4}^{+}$ and pH were measured shipboard. Hydrogen sulfide and ${NH}_{4}^{+}$ were determined by colorimetric methods using a continuous flow analyzer (Seal) and pH by using a portable Metrohm pH meter. Aliquots for later shore-based analyses of Cl and ${SO}_{4}^{2-}$ by a Metrohm IC system (ion chromatography), and of Na, Ca, K, Mg, Si, and Fe by a Thermo IRIS ICP-OES (inductively coupled plasma optical emission spectrometry), were stored in HDPE bottles at about 4°C until analysis. The samples for ICP-OES analyses were stored in acid cleaned bottles and acidified by ultrapure nitric acid to 3 vol %. Relative SDs for both, IC and ICP-OES measurements were <5%. In HFs, quantitative removal of Mg occurs rapidly during SW-rock interaction and a 0 Mg fluid is assumed to exit the chimneys (Mottl and Holland, 1978). SW entrainment during sampling of the focused high-temperature Bruse chimney was thus corrected by extrapolation of the measured concentration through 0 Mg. Measurements of the diffusely venting fluids sampled at Soria Moria Vent Field are reported as uncorrected values.

### Subsampling of Chimney Walls

Once shipboard, subsamples of the chimney fragments from the exterior toward the inner flow channel were taken using sterile scalpels. First, outer layers (up to approximately 1 mm into the chimney wall) were scraped off and sampled. Then the remains of this outer material on the chimney were removed (using a scalpel), and subsampling of the next layer continued. Subsequent subsamples were taken accordingly, throughout the first few centimeters of the chimney wall. In addition, we sampled the black, porous minerals found further inside the chimney walls (10–15 cm from outer surface). Replicates represent samples from the same chimney layer, but from which DNA was extracted independently. At the sites of sampling, the chimney walls were 10–20 cm thick (distance from outer surface to inner fluid-flow channel). Subsamples were placed in cryo-vials that were immediately frozen in liquid nitrogen and then stored at -80°C until further processing onshore. Information about subsamples, from which we were able to amplify the 16S rRNA gene, is shown in **Table 1**.

**Table 1:** List of samples from which 16S rRNA amplicons were obtained.

Sample name	Distance from outer surface	# Raw reads	# Clean reads	DNA content (ng/g chimney)	Estimated cell numbers (cells/g)
S1.1	0–1 mm	927	637	8228.6	3.3 × 10^9^
S1.2		2821	2002	4965.5	2.0 × 10^9^
S1.3		993	610	ND	ND
S2.1	1–5 mm	843	592	1590.0	6.3 × 10^8^
S2.2		6271	4898	775.0	3.1 × 10^8^
S3.1	5–10 mm	440	300	181.5	7.3 × 10^7^
S3.2		5808	4242	200.0	8.0 × 10^7^
S3.3		1729	986	ND	ND
S4.1	17–20 mm	2181	1273	24.0	9.6 × 10^6^
S4.2		881	535	5.6	2.2 × 10^6^
S4.3		2706	1850	ND	ND
S5.1	27–30 mm	1015	838	5.8	2.3 × 10^6^
S5.2		15088	11935	14.7	5.9 × 10^6^
S5.3		11651	9286	ND	ND
B1.1	0–1 mm	22773	21249	10285.7	4.1 × 10^9^
B1.2		25924	23879	10731.7	4.3 × 10^9^
B2.1	1–5 mm	27108	8223	3763.8	1.5 × 10^9^
B2.2		18252	16906	6344.8	2.5 × 10^9^
B3.1	5–10 mm	22075	20904	1760.3	7.0 × 10^8^
B3.2		30891	28886	1844.2	7.4 × 10^8^
B4.1	17–20 mm	30423	29265	54.3	2.2 × 10^7^
B4.2		36815	34947	72.5	2.9 × 10^7^
B5.1	10 cm	21712	17741	392.5	1.6 × 10^8^
B5.2		21157	19743	303.2	1.2 × 10^8^


*S1–S5 were taken from the Soria Moria chimney and B1–B5 from the Bruse chimney. Duplicate or triplicate DNA extractions were performed for each sample and analyses separately (indicated by the last number in each sample name). Cell number estimates were calculated from the DNA content assuming a mean value of 2.5 fg DNA cell^-*1*^ (Button and Robertson, 2001). ND, not determined.*

### DNA Extraction and PCR

Total DNA was extracted using the FastDNA spinkit for soil (MP Biomedicals). Amplification of 16S rRNA genes was performed as previously described (Roalkvam et al., 2011), using a two-step PCR approach. In short, a first PCR step was performed with up to 20 ng template in 25–33 cycle reactions using primers universal for Archaea and Bacteria—Un787f (5′-ATTAGATACCCNGGTAG) and Un1392r (5′-ACGGGCGGTGWGTRC). Reactions were run in triplicates in order to minimize PCR bias. PCR products were pooled and rinsed with the MinElute PCR purification kit (Qiagen) prior to a five-cycle second PCR step, where approximately 20 ng of PCR product was used as template, applying GS FLX Titanium fusion primers and a sample specific barcode attached to the forward primer. Sequencing was performed at The Norwegian High-Throughput Sequencing Centre and at Microsynth (Switzerland) using the Roche/454 GS-FLX Titanium system.

### Filtering, Taxonomic Analyses and Operational Taxonomic Unit (OTU) Clustering

Amplicons were filtered (**Table 1**) and clustered into OTUs using MOTHUR (version 1.33) (Schloss et al., 2009). Low quality reads were removed with PYRONOISE (Quince et al., 2011) as implemented in MOTHUR (command “shhh.flows”). Reads shorter than 362 bp, as well as chimeras detected by UCHIME were discarded. OTUs were picked at the 97% level using the average neighbor algorithm on uncorrected pairwise distances.

Taxonomic assignments were performed in MacQIIME (version 1.9) (Caporaso et al., 2010) using the uclust method (Edgar, 2010) and with SILVA as reference database (release 111)^1^.

### Modeling

Mixing modeling was performed as described previously (Amend et al., 2011; Dahle et al., 2015). In short the REACT module of the Geochemists Workbench^TM^ (GWB) software package was used to simulate a stepwise mixing of 1 kg of HF with 5000 kg of SW. The reaction path mimics the incremental titration of small aliquots of cold SW into hot vent fluids. For each step the temperature and chemical speciation was evaluated. Minerals were not allowed to precipitate during mixing, redox-reactions were prohibited, while acid-base reactions were allowed to reach equilibrium. The GWB analyses used the thermo.com thermodynamic database modified to relevant temperatures and pressures using the “subcrt” command in the R package CHNOSZ (Dick, 2008), which calculates the standard thermodynamic properties of species and reactions as a function of temperature and pressure, and is modeled after the functionality of the SUPCRT92 package (Johnson et al., 1992). Gibbs energies were calculated from the equation

[1] $$\Delta G_{r}=\Delta G_{r}^{0}+RT\,\;ln\,\;Q$$

where ΔG*_r_* is the Gibbs energy of the reaction (in kJ mole^-1^), ΔG*_r_*^0^ is the standard Gibbs energy of the reaction at the relevant temperature and pressure (in kJ mole^-1^), *R* is the gas constant (0.00831 kJ K^-1^ mole^-1^), *T* is the temperature in Kelvin, and *Q* is the activity product of reaction *r* defined as

[2] $$Q=\Pi {a_{i}}^{\nu_{i}}\,$$

where a*_i_* is the activity of the *i*th chemical species, and *ν_i_* is the stoichiometric reaction coefficient, which is positive for products and negative for reactants.

Models of relative abundances of functional groups of primary producers were based on modeled energy landscapes, as described by Dahle et al. (2015). In model simulations, each predefined functional group (**Table 2**) was allowed to “spend” a low number of electrons (10 nanomoles) at a time on acquiring energy from their corresponding redox-reaction. For organisms having the ability to grow on more than one redox-reaction [i.e., sulfide and hydrogen oxidizers (SHOs)], the most exergonic redox-reaction was preferred. Concentrations of each substrate were continuously adjusted by removing the number of moles consumed in each electron transfer. Energy could only be acquired from a reaction if the concentration of all substrates was above 0. The “spending” of electrons continued iteratively until all limiting substrates were consumed. Finally, the relative abundance of each functional group was set to be equal to the relative amount of energy acquired by that group [see (Dahle et al., 2015) for details]. In essence, this is similar to equating relative densities of energy in the energy-models to relative abundances of functional groups. The difference is that in the energy-models each redox reaction is considered independently, whereas in the community models each molecule of substrate can only be consumed once. For example, in the energy-models the potential energy available from aerobic sulfide oxidation is calculated independently from the energy available from aerobic methane oxidation. In the community models, however, the energy acquired by aerobic sulfide oxidizers will depend on the amount of oxygen consumed by aerobic methane oxidizers. Importantly, our models only considered primary producers, which for our purpose was defined as organisms acquiring energy utilizing substrates readily available in HFs and SW. According to this definition, organism utilizing metabolic products of other organisms was not considered as primary producers, but as part of a higher trophic level.

**Table 2:** Overview of chemical processes and definition of functional groups according to redox-reactions used as energy source.

Functional group	Short name	Process	Redox-reaction
Sulfur and hydrogen oxidizers	SHO	Sulfide oxidation with oxygen	H_2_S + 2O_2_ → HSO_4_^-^ + H^+^ HS^-^ + 2O_2_ → HSO_4_^-^
		Hydrogen oxidation	2H_2_ + O_2_ → 2H_2_O
Methane oxidizers	MO	Methane oxidation (aerobic)	CH_4_ + 2O_2_ → CO_2_ + 2H_2_O
Ammonium oxidizers	AMO	Ammonium oxidation	NH_4_ + 2O_2_ → ${NO}_{3}^{-}$ + H_2_O + 2H
Iron oxidizers	FEO	Iron oxidation	4Fe^2+^ + O_2_ + 10H_2_O → 4Fe(OH)_3_ + 8H^+^
Anaerobic methane oxidizers	AOM	Methane oxidation (anaerobic)	CH_4_ + ${SO}_{4}^{2-}$ + 2H^+^ → CO_2_ + H_2_S + 2H_2_O
Methanogens	MET	Methanogenesis	4H_2_ + CO_2_ → CH_4_ + 2H_2_O 4H_2_ + HCO_3_^-^ + H^+^ → CH_4_ + 3H_2_O
Sulfate reducers	SR	Sulfate reduction	4H_2_ + ${SO}_{4}^{2-}$ + 2H^+^ → H_2_S + 4H_2_O


*Compounds in the redox-reactions are in the aqueous phase, except for Fe(OH)_*3*_ which represents the mineral ferrihydrite.*

### Heatmaps, Clustering, Functional Assignments, and Principle Component Analyses

Heatmaps, showing relative abundances of different taxa, were made using the “heatmap.2” function in the GPLOTS R package (Warnes et al., 2011). Cluster analyses were based on a distance matrix generated from the OTU-table using Bray-Curtis Distances (BCDs). Ward clustering was performed with squared dissimilarities before cluster updating (Murtagh and Legendre, 2014) using VEGAN (command “hclust,” method “ward.D2”). 16S rRNA gene sequences were assigned to functional groups based on taxonomic affiliations (see results section). Based on the functional assignments, we constructed a table of relative abundances of functional groups, excluding putative organotrophs and functionally unassigned organisms. The table was subject to Hellinger transformation before ordination diagrams were constructed using Principle Component Analyses (PCA) “rda” command of the VEGAN R package (Oksanen et al., 2011).

### Deposition of Sequence Data

Raw sequence data have been submitted to the Sequence Read Archive under the accession numbers SRR5099177-SRR5099178 and SRR5099180-SRR5099193.

## Results

### Fluid Chemistry and Modeling

**FIGURE 2:**
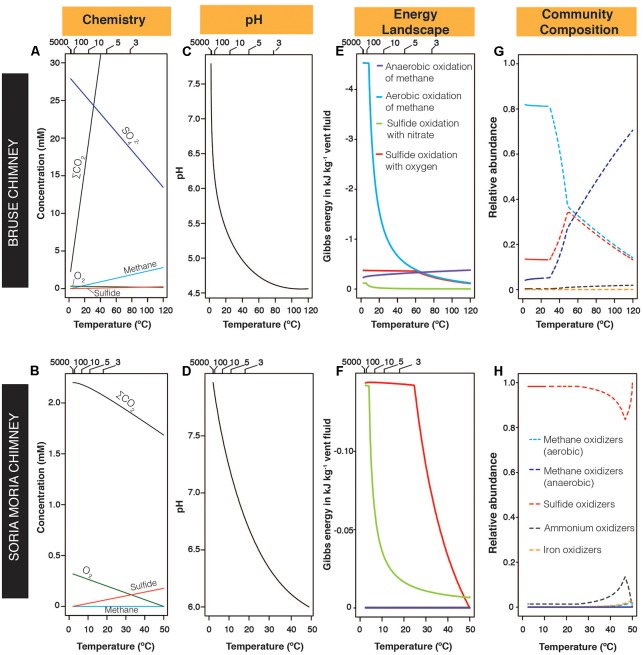
Modeled chemical gradients, energy landscapes, and microbial communities in the Bruse chimney and the Soria Moria chimney at different temperatures. **(A,B)** Modeled concentrations of selected chemical species. Sulfate concentrations in the Soria Moria chimney are not shown, but were in the range of 27–28 mM. **(C,D)** Modeled changes in pH across the two chimneys. **(E,F)** Modeled energy landscapes based on reactions given in **Table 2**. For clarity, graphs are not shown for reactions where Gibbs energies are close to 0 for all temperatures considered in both chimneys. **(G,H)** Modeled relative abundances of functional groups defined in **Table 2**. For clarity, graphs are not shown for functional groups with relative abundances <1% for all considered temperatures in both chimneys. Upper horizontal axes in **(A–F)** indicate seawater:hydrothermal-fluid mixing ratios. Legends in **(E)** also apply for **(F)**, legends in **(G)** also apply for **(H)**.

The chemical composition of fluids from the Soria Moria chimney and the Bruse chimney varied considerably (**Table 3**). In particular, CH_4_:H_2_S ratios were 5927 times higher in the Bruse chimney than in the Soria Moria chimney. Mixing modeling combined with thermodynamic calculations considering selected redox-reactions (**Table 2**), indicated highly different energy landscapes in the two chimney walls (**Figure 2**): In the Soria Moria chimney, sulfide oxidation was predicted to be the dominant energy source. Energy densities of sulfide oxidation was higher in the Bruse chimney than in the Soria Moria chimney, nevertheless, aerobic methane oxidation (<∼60°C) and anaerobic methane oxidation (>∼60°C) dominated as energy-sources in the Bruse chimney. Energy landscapes were transformed into microbial community composition models, only considering pre-defined functional groups of organisms (**Table 2**). The community structure models suggested a dominance of SHOs throughout the entire Soria Moria chimney wall. In the inner sections, the SHOs are predicted to obtain energy mainly using nitrate as an electron acceptor. Toward the chimney surface, the abundance of SHOs drops when oxygen concentrations become higher, as long as it remains limiting for all functional groups. Further toward the surface, electron donors become limiting for all functional groups except for SHO and this functional group increases to a relative abundance of nearly 1.0. Community modeling from the Bruse chimney, predicted a dominance of anaerobic methane oxidizers in the inner parts of the chimney and at temperatures above around 60°C, whereas at lower temperatures (i.e., toward the outer parts), aerobic methane oxidizers dominated. However, sulfide oxidizers were also predicted to be relatively abundant throughout the Bruse chimney wall. Energy availabilities in reactions involving O_2_ or ${NO}_{3}^{-}$ as electron acceptors reached a plateau at high SW:HF mixing ratios. These plateaus occur at mixing ratios where the electron acceptor is no longer limiting (not shown).

**Table 3:** Chemical composition of vent fluids and seawater used in this study.

Sample	T (°C)	pH	Mg	Na^+^	Ca^2+^	K^+^	Fe^2+^ (μM)	Cl^-^	Si	CH_4_ (μm)	H_2_S	H_2_ (μm)	ΣCO_2_ (mm)	${NH}_{4}^{+}$(μM)	${SO}_{4}^{2-}$	${NO}_{3}^{-}$ (μM)
Bruse	229	4.7	15.1	377	27.9	34.3	7.3	469	8.9	5400	0.41	10	174	33.0	ND	ND
Soria Moria	50	6.0	50	432	11.9	12.5	4.0	521	0.8	0.4	0.18	0.30	1.7	5.8	27.3	13.3
Seawater^∗^	1	7.9	52	442	10.2	9.8	ND	545	ND	ND	ND	ND	2.3	ND	27.9	12.0


*^∗^The temperature of ambient bottom seawater is -0.4°C at both vent fields, but was set to 1°C in the models. In addition to the listed chemical species, seawater also includes 405 μm O_*2*_. For the Bruse chimney, reported values are endmember values after Mg correction, except for the Mg concentration itself which indicates the concentration in the sampled fluids. Fe^*2*+^, NH_*4*_^+^ and NO_*3*_^-^ are given in micromolar (μM), CH_*4*_ and H_*2*_ in micromolal (μm), and CO_*2*_ in millimolal (mm). All other concentrations are given in millimolar (mM). ND, not detected.*

### Observed Composition and Diversity of Microbial Communities in Chimney Wall Transects

**FIGURE 3:**
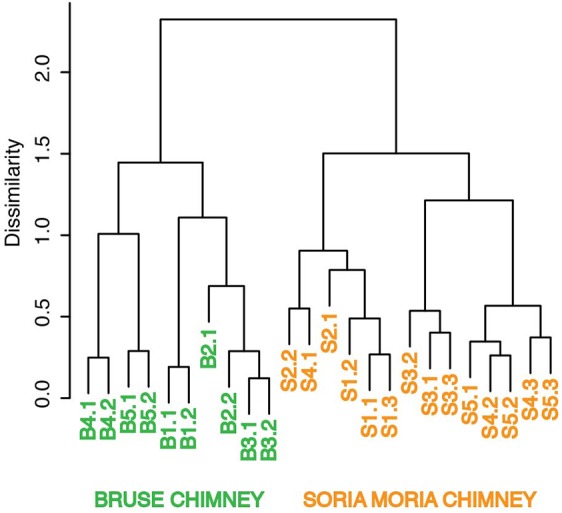
Ward’s minimum variance clustering of samples from Soria Moria- and Bruse chimney walls. Clustering was based on Bray-Curtis distances of relative abundances of OTUs. Sample names correspond to the short names given in **Table 1**.

**FIGURE 4:**
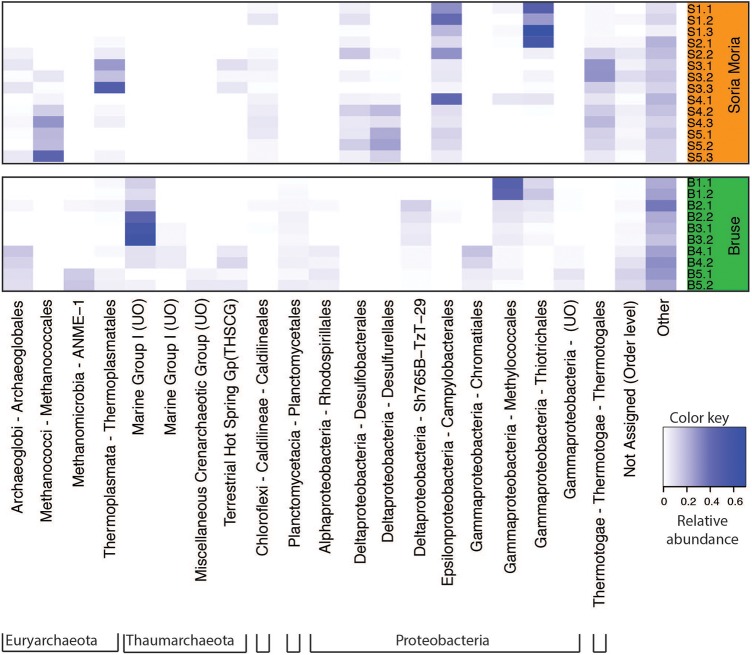
Heatmap of relative abundances of dominating taxa (at least one sample with relative abundance >1%) at the order level. Sample names correspond to the short names given in **Table 1**. UO, unnamed order.

Clustering based on OTU level BCDs revealed a distinct clustering according to which chimney the samples were collected from. In general, subsamples from the same chimney region clustered together. However, one notable exception is sample S4.1, which clustered together with S2.2 (**Figure 3**). Samples from the two chimneys were also highly dissimilar on the taxonomic level (**Figure 4**; Supplementary Material). The outer sections of the Soria Moria chimney (layers S1 and S2) were dominated by relatives of aerobic sulfur oxidizers within the genus *Sulfurimonas* (class Epsilonproteobacteria) and the family Thiotrichaceae (class Gammaproteobacteria). Further inside the wall of this chimney (layer S3), the communities were dominated by relatives of organotrophic members of the genus *Kosmotoga* (order Thermotogales) and members of the Deep Sea Hydrothermal Vent Group II (DHVEG-2) (order Thermoplasmatales). The innermost sections (layers S4 and S5), were dominated by relatives of sulfate-reducing members of the genus *Hippea* (class Deltaproteobacteria) and methanogenic members of the genus *Methanococcus* (order Methanococcales). The outer section of the Bruse chimney (layer B1) was dominated by relatives of aerobic methane oxidizers of the Hyd24-01 clade (order Methylococcales). Further inside this chimney, one layer (layer B2) was dominated by OTUs assigned to Marine Gr 1 Thaumarchaeota and which were related to cultured members of *Nitrosopumilus* (92–97% 16S rRNA gene sequence identity with “*Candidatus* Nitrosopumilus sp.” strains NF5 and D3C (Bayer et al., 2016)). Deeper sections of the Bruse chimney (layers B4 and B5) were dominated by relatives of anaerobic methane oxidizers within the ANME-1 clade (class Methanomicrobia) and SRBs within the genus *Archaeoglobus*.

### Distribution of Functional Groups in Environmental Samples and Model Comparisons

**FIGURE 5:**
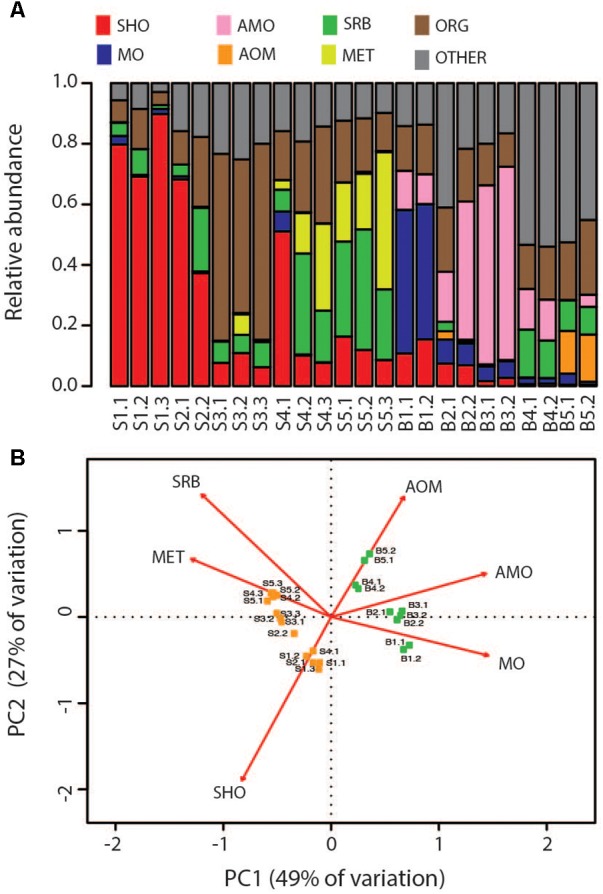
Comparison of the Bruse chimney and Soria Moria chimney in terms of the functional groups of microorganisms they host. Functional assignments were performed based on taxonomic information (see Supplementary Material). Abbreviations of functional groups are the same as in **Table 2**. **(A)** Distribution of functional groups in each sample. **(B)** Ordination diagram in two dimensions from Principal Component Analyses based relative abundances of functional groups after removal of organotrophs and metabolically unassigned organisms. The ordination is drawn as a biplot where samples are represented as dots and functional groups by arrows. Eigenvectors are scaled to unit length and distances among objects and the biplot are approximations of their Euclidian distance in multidimensional space. Arrows point in the direction of maximum variance of relative abundance of respective functional groups. Arrow lengths correspond to the contribution to variation in community composition between samples. Sample names correspond to the short names given in **Table 1**.

**FIGURE 6:**
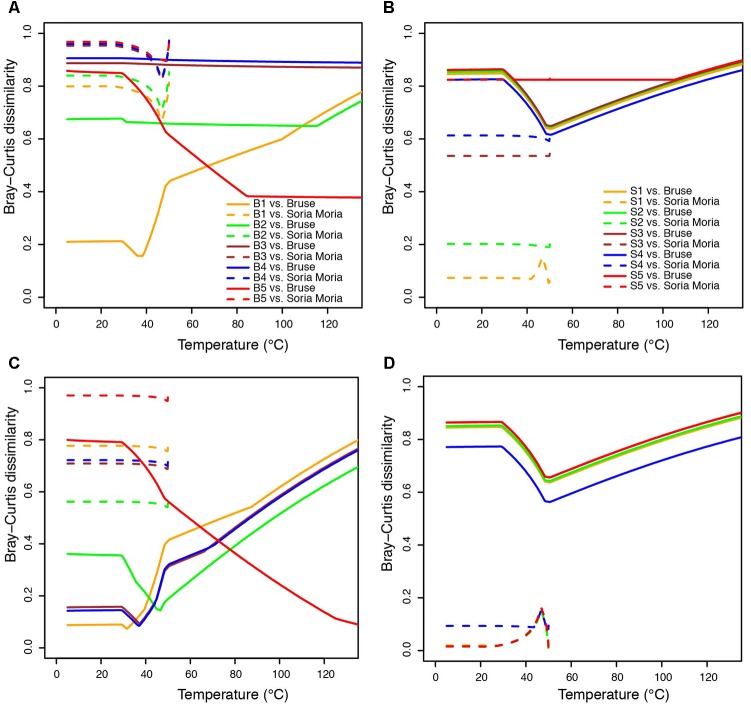
Bray-Curtis Distances (BCDs) between modeled communities and relative abundances among functional groups of primary producers in environmental samples—i.e., only considering organisms assigned to the functional groups defined in **Table 2**. Functional assignments of detected taxa are shown in **Figure 5**. Each line shows BCD values in comparisons between one sample and all modeled communities in one chimney. Low BCD values indicate good fits between models and sampled communities. **(A)** BCDs between sampled communities in the Bruse chimney and modeled communities from the Bruse chimney and the Soria Moria chimney. **(B)** BCDs between sampled communities in the Soria Moria chimney and modeled communities in the Bruse chimney and the Soria Moria chimney. **(C,D)** same as **(A,B)**, respectively, except that methanogens and sulfate reducers are not considered as primary producers and that Marine Gr. 1 are considered as aerobic methane oxidizers instead of ammonium oxidizers. Legends in **(A)** also apply to **(C)**. Legends in **(B)** also apply to **(D)**.

In order to compare modeled microbial communities with communities in environmental samples, it was necessary to assign 16S rRNA genes to metabolic functions. This was done based on current knowledge about the energy metabolism of organisms within the detected taxa (Supplementary Material). Since 16S rRNA genes are not necessarily good markers for functional traits, such assignments are uncertain. A complicating issue is that many organisms can choose between different energy metabolisms. For example, SRBs are facultative autotrophs, and may have the ability to grow as lithotrophs (i.e., with hydrogen as electron donor) or organotrophs (e.g., by using various volatile fatty acids as electron donors). Nevertheless, major trends in the distribution of broad metabolic categories, were in agreement with modeling (**Figure 5**): samples from the Soria Moria chimney were associated by high relative abundances of SHOs whereas samples from the Bruse chimney were associated with high relative abundances of methane oxidizers (AOM, MO). Moreover, surface layers of the Bruse chimney were associated with putative aerobic methane oxidizers, whereas deeper layers were associated with putative ammonium oxidizers and putative anaerobic methane oxidizers. The models also predicted low relative abundances (<0.04%) of SRBs growing with H_2_ as electron donor in throughout the chimney walls. Yet, SRB were detected with relative abundances of more than 3% in most samples and reached relative abundances of up to 40% in the inner sections of the Soria Moria Chimney (layer S5). BCDs were used to quantitatively compare modeled and observed communities—i.e., the distribution of putative primary producers in each chimney layer was compared to modeled communities at different temperatures both from the Bruse Chimney and the Soria Moria Chimney (**Figure 6**). Comparisons were done with different assumptions during functional assignments: In **Figures 6A,B** all SRBs and methanogens are considered as primary producers utilizing H_2_ whereas members of Marine Gr. 1 are considered as primary producers utilizing ammonium. In **Figures 6C,D** it was assumed that members of Marine Gr. 1 are aerobic methanotrophs and that methanogens as well as SRBs are not primary producers utilizing electron donors originating from HFs, but grow on metabolic products of fermentative organisms. The support for these assumptions is further discussed in the discussion section. In general, BCDs between modeled and observed communities where high, but dropped when observed communities were compared to modeling results from the same environmental setting. For example, low BCD values were observed between layer S1 versus modeling at low temperatures at the Soria Moria Chimney, layer B1 versus modeling at low temperatures at the Bruse Chimney and layer B5 versus modeling at high temperatures from the Bruse Chimney. However, in **Figures 6A,B**, poor fits to models were observed for inner parts from the Soria Moria Chimney and some of the intermediate parts of the Bruse Chimney. Yet, also these samples were largely in agreement with modeling under the assumption used in **Figures 6C,D**. Taken together, we found, with some notable exceptions, an overall good correspondence between distributions of primary producers in different chimneys and different parts of the same chimney versus energy-based modeling.

## Discussion

In this study, we used a combination of taxonomic profiling, functional assessments, geochemical modeling, and thermodynamic considerations to investigate what effect shifting energy landscapes in hydrothermal chimneys have on microbial community composition. Consistent with previous findings (Dahle et al., 2015), we provide evidence for a tight coupling between variations in energy landscapes and variations in the distribution of functional groups of organisms, at least for the outer parts and high-temperature inner parts of the chimneys in hydrothermal systems along AMOR. However, we also observed some discrepancies between models and field studies. Possible reasons for these discrepancies are discussed below together with an evaluation of sampling strategy and energy assessments.

### The Fluid Chemistry of the Soria Moria and Bruse Chimneys

The Bruse chimney vented visually observed focused flow fluids with a temperature of 229°C. High temperature venting suggests that sub-seafloor mixing with SW is not taking place. Thus, the Mg content was used to corrected for SW entrainment during sampling. The Soria Moria chimney show diffuse flow characteristics venting fluids with a temperature of 50°C, which imply sub-seafloor mixing of the endmember vent fluid with circulating SW. Magnesium concentrations of 50 mmol/l in the Soria Moria chimney fluid indicate a vent fluid contribution of about 4% in these fluids. A vent fluid input in the same range is shown by other alkali and alkaline earth metals as well as by the Cl concentration in comparison to background SW. However, it cannot be excluded that some ambient SW was collected during sampling of the Soria Moria HFs. The implications of this for the community composition modeling can be expected to be of minor importance as these models consider relative abundances of functional groups, which ultimately depend on ratios of chemical compounds and not absolute concentrations. In this respect it is also worth emphasizing that high ratios of H_2_S:CH_4_ and H_2_S:H_2_ observed in the present study of the Soria Mora chimney are comparable to ratios previously observed in high temperature fluids from the Soria Moria Vent Field (Baumberger, 2011; Dahle et al., 2015).

### Evaluation of Sampling Strategy

The sampling of hydrothermal chimneys in order to detect variations in microbial community structure down to millimeter scales is challenging. In the present study, we did a subsampling by scraping off subsequent chimney layers using a scalpel. However, with this strategy some cross-contamination from DNA-rich outer layers to DNA-poorer inner layers (**Table 1**) during sampling is difficult to avoid. Some variations between replicates from the same layer were also expected as they may be associated with different porosities, fluid flow patterns and hence SW:HF mixing ratios. The observation that samples from different layers of the chimney in some cases cluster closely together in cluster analyses [i.e., samples S4.1 and S2.2 (**Figure 3**)] was therefore expected. However, in most cases samples from the same or adjacent layers clustered together in cluster analyses and PCA (**Figures 3**, **5B**), showing that cross-contamination between different layers was limited.

### Comparison Between Models and Observations

Community composition models based on thermodynamic considerations (**Figure 2**), suggested that the Soria Moria chimney and the Bruse chimney could be distinguished mainly by relative abundances of aerobic methane oxidizers, anaerobic methane oxidizers and SHOs. Moreover, in the Bruse chimney, aerobic methane oxidizers were predicted to predominate in outer sections, whereas anaerobic methane oxidizers were predicted to predominate in inner sections. These trends are consistent with the distribution of functional groups of primary producers as assessed from 16S rRNA gene sequencing (**Figure 5**), which demonstrates that the models have predictive power, and provide evidence for a tight coupling between energy landscapes and distribution of primary producers. Discrepancies between models and observations (most evident in layers B3, B4, and S5 in **Figures 6A,B**), have several possible explanations: (1) A fundamental assumption in the community models—i.e., that there is a direct coupling between relative energy densities and relative abundances of functional groups—may not always be valid. (2) Energy landscapes derived from fluid mixing models, may in some cases give results that are far from reality. For example, abiotic processes, such as abiotic reduction of O_2_ or water-rock reactions, are not considered. Neither do the models consider how the metabolic activities of organisms affect energy landscapes. (3) Functional assignments based on 16S rRNA gene sequence analyses may not be correct (4) Biases introduced in the analyses of environmental samples—i.e., during DNA extraction and PCR—may have a large impact on our characterization of microbial community composition in the chimneys. For example, major metabolic groups may have been missed if they fail to be amplified with the selected pair of primers.

With this in mind, it is difficult to evaluate whether discrepancies between models and observed communities is a results of erroneous model predictions or incorrect reconstruction of the analyzed communities. Interestingly, however, major discrepancies in **Figures 6A,B** were highly sensitive to assumptions about SRBs, methanogens, and Marine Gr. 1 (**Figures 6C,D**). Best fits between models and observations were observed under the assumption that detected members of Marine Gr. 1 are aerobic methanotrophs and that SRBs and methanogens are not primary producers. So far, no member of Marine Gr. 1 has ever been isolated from a hydrothermal system making it difficult to infer what metabolic role they have in these settings. Our knowledge about the functional diversity of organisms within this taxonomic group is also in general limited (Bayer et al., 2016). Nevertheless, it is interesting to note that close relatives of *Nitrosopumilus* have previously been found to dominate on the outer wall of chimneys from the Mothra Vent Field (Juan de Fuca Ridge) (Schrenk et al., 2003), which also have venting fluids with high methane content (Lin et al., 2016). Hence, the possibility that members of Marine Gr. 1 detected in the current study are methanotrophs, as suggested by modeling, cannot be excluded. The assumption that SRBs and methanogens are not primary producers also seems plausible. In the chimney walls analyzed in the present study, we detected high abundances of putative organotrophs, primarily members of Thermotogales (genus *Kosmotoga*) and Thermoplasmatales (clade DHVEG-2). In the Soria Moria chimney, members of *Kosmotoga* predominated in a distinct layer some millimeters away from dense communities of sulfide oxidizers on the chimney surface. It seems therefore likely that they grow on organic carbon produced by primary production. Cultured *Kosmotoga* strains grow by fermentation and produce acetic acid and hydrogen (DiPippo et al., 2009). If these metabolites diffuse further into the deeper parts of the chimney, this could explain the high abundances of sulfate reducing Deltaproteobacteria and methanogenic *Methanococcus* in layers S4 and S5. Such a relationship is similar to what has been observed at the Endeavour hydrothermal field where hyperthermophilic heterotrophs, such as *Thermococcus* species, have been proposed to support the growth of methanogens through H_2_ syntrophy (Ver Eecke et al., 2012). Also, the capacity for H_2_ syntrophy in diffuse vent fluid from Axial Seamount was shown by Topcuoglu et al. (2016). Syntrophic SRBs and methanogens should not be considered as primary producers, as they are utilizing electron donors not readily available in the HFs, but are products of other organisms metabolism. This illustrates that even though energy landscapes modeled from simple mixing modeling, as done in the present study, can be used to infer broad patterns in the distribution of functional groups, actual energy landscapes are probably also shaped by complex biotic and abiotic processes, including the microorganisms own metabolic activity.

Taken together, even though we observe a poor fit between modeled and observed communities in some of our samples, this does not necessarily imply that there is no strong connection between energy densities and distributions of functional groups also in these cases. Rather, a misfit between models and observations may be an indicator of erroneous metabolic assignments of detected taxa or an indication that the metabolic activities of microorganisms largely modulate energy landscapes. Development of population dynamic models may be a fruitful approach to obtain community structure models with improved predictive power and which take entire communities into account instead of only primary producers. Such models may be formulated as differential equations explicitly expressing the competition for substrates shared by several metabolic groups of organisms and the flow of energy through food webs. Comparing such models with environmental analyses involving shotgun metagenomics, transcriptomics, and rate measurements seems to be a promising strategy for further deciphering of the connections between energy availability and microbial community structure in hydrothermal systems.

## Author Contributions

HD conceived and directed the study, did the modeling, analyzed 16S rRNA reads, and wrote the paper. SLMB obtained the 16S rRNA gene amplicons. HD, SLMB, TB, RP, IT, and IS obtained the samples. TB and IT measured the chemical composition of hydrothermal fluids. All coauthors edited the manuscript.

## Conflict of Interest Statement

The authors declare that the research was conducted in the absence of any commercial or financial relationships that could be construed as a potential conflict of interest.

## Footnotes

1 https://www.arb-silva.de/
