# Supplementary material for: Energy Landscapes in Hydrothermal Chimneys Shape Distributions of Primary Producers
Source: Front Microbiol. 2018 Jul 16;9:1570. doi: 10.3389/fmicb.2018.01570 (PMC6055050; doi:10.3389/fmicb.2018.01570)
Supplement: Supplementary file 1 [file Data_Sheet_1.DOCX]

Supplementary Material

Differences in energy landscapes in cross sections of hydrothermal chimneys reflect differences in what microbial communities they host.

Håkon Dahle^*^, Sven Le Moine Bauer, Tamara Baumberger, Runar Stokke, Rolf Birger Pedersen, Ingunn Hindenes Thorseth, Ida Helene Steen.

*** Correspondence:** Corresponding Author: Hakon.Dahle@uib.no

# Supplementary Data

Supplementary Table 1 – Functional assignments of selected taxa. Average relative abundances across all samples from both chimneys are reported for selected subtaxa within each taxon. Numbers in parentheses indicate standard deviations.

| Taxon | Dominating subtaxa | Average relative abundance within taxon in % (sd) | Functional assignment |
| --- | --- | --- | --- |
| Epsilonproteobacteria (class) | *Sulfurimonas* (genus)  *Sulfurovum* (genus)  *Campylobacter* (genus)  *Nitratifractor* (genus)  *Sulfurospirillum* (genus)  *Arcobacter* (genus)  *Helicobacter* (genus) | 68 (22)  9.5 (13)  10 (15)  4.6 (8.7)  4.6 (6.2)  3.0 (8.0)  1.1 (4.2) | SHO  SHO  SHO  SHO  SHO  SHO  SHO |
| Thiotrichales (order) | Thiotrichaceae (family)  Piscirickettsiaceae (family)  Francisellaceae (family) | 66 (32)  30 (29)  2.6 (9.8) | SHO  SHO  SHO |
| (continued) |  |  |  |
| Methylococcales (order) | Hyd24-01 (family)  Unassigned Methylococcales  IheB2-23 (family)  *Methylothermus* (genus)  *Methylomonas* (genus)  *Methylobacter* (genus) | 69 (34)  24 (33)  3.7 (6.5)  1.4 (4.2)  1.3 (4.4)  0.8 (3.6) | MO  MO  MO  MO  MO  MO |
| Marine Gr. 1 (class)^*^ | Unassigned Marine Gr. 1  *Nitrosopumilus* (genus) | 94 (13)  5.5 (13) | AMO  AMO |
| Methanococci | *Methanococcus* (genus) | 100 (0) | MET |
| ANME-1 (order) |  |  | AnMO |
| ANME-2c (family) |  |  | AnMO |
| Archaeoglobales (order) | *Geoglobus* (genus)  *Archaeoglobus* (genus)  Unassigned Archaeoglobaceae (family) | 48 (46)  9.5 (15)  41 (38) | SR  SR |
| Thermodesulfobacteriales  (order) | Unassigned Thermodesulfobacteriaceae (family) | 100 (0) | SR |
| Aquificales (order) | *Sulfurihydrogenibium* (genus)  *Desulfurobacterium* (genus)  *Hydrogenothermus* (genus)  *Hydrogenivirga* (genus)  *Thermosulfidibacter* (genus)  *Persephonella* (genus)  Unassigned Hydrogenothermaceae | 30 (43)  12 (29)  1.6 (3.6)  23 (38)  23 (38)  1.2 (2.3)  6.7 (21) | SHO  OTHER  SHO  SHO  OTHER  SHO  SHO |
| (continued) |  |  |  |
| Desulfobacterales (order) | *Desulfobacterium* (genus)  *SEEP-SRB1* (genus)  *Desulfobulbus* (genus)  *SEEP-SRB2* (genus)  *Desulfocapsa* (genus)  *Nitrospina* (genus)  *Desulfurivibrio* (genus)  *SEEP-SRB4* (genus) | 56 (45)  18 (24)  1.6 (3.4)  4.7 (10)  3.5 (12)  14 (20)  1.7 (6.2)  1.1 (3.0) | SR  SR  SR  SR  SR  OTHER OTHER  SR  SR |
| Desulfurellales (order) | *Hippea* (genus) | 100 (0) | SR |
| Thermotogales (order) | *Kosmotoga* (genus)  *Thermotoga* (genus)  *Fervidobacterium* (genus)  Unclassified Thermotogaceae (family) | 82 (39)  14 (31)  2.3 (9.5)  1.5 (5) | ORG  ORG  ORG  ORG |
| Thermoplasmata (class) | DHVEG-2 (family)  AMOS1A-4113-D04 (family)  VC2.1_Arc6 (family)  Other Thermoplasmata | 41 (37)  26 (32)  8 (15)  25 | ORG  ORG  ORG  ORG |
| Chloroflexi (class) | Caldilineaceae (family)  SAR202_clade (order)  *Anaerolineaceae* (genus)  Other Chloroflexi | 62 (41)  28 (33)  2.2 (5)  7.8 | ORG  ORG  ORG  ORG |
| Terrestrial_Hot_Spring_Gp(THSCG) (class within Thaumarchaeota) | Unclassified THSCG | 100 (0) | ORG |
| Miscellaneous_Crenarchaeotic_  Group (MCG)  (continued) | Unclassified MCG | 100 (0) | ORG |
| Planctomycetes (Phylum) | *Planctomyces* (genus)  OM190 (class)  *Phycishaera* (genus)  08D2Z94_hypersaline_microbial_mat_group  *Rhodopirellula* (genus)  Other Planctomyctetes | 20 (21)  27 (28)  8 (22)  3.1 (11)  2.2 (4)  39.7 | ORG  ORG  ORG  ORG  ORG  ORG |
| Bacteroidetes (Phylum) | Unclassified VC2.1_Bac22 (class)  *Lutibacter* (genus)  *Muricauda* (genus)  *Persicobacter* (genus)  *Maritimonas* (genus)  Other Bacteroidetes | 33 (31)  12 (14)  4 (9)  7 (14)  2 (3)  42 | ORG  ORG  ORG  ORG  ORG  ORG |
| Thermococci (Class) | *Thermococcus* (genus) | 100 (0) | ORG |

* In Fig. 6 C-D, members of Marine Gr.1 were assumed to be aerobic methanotrophs (MO).

**
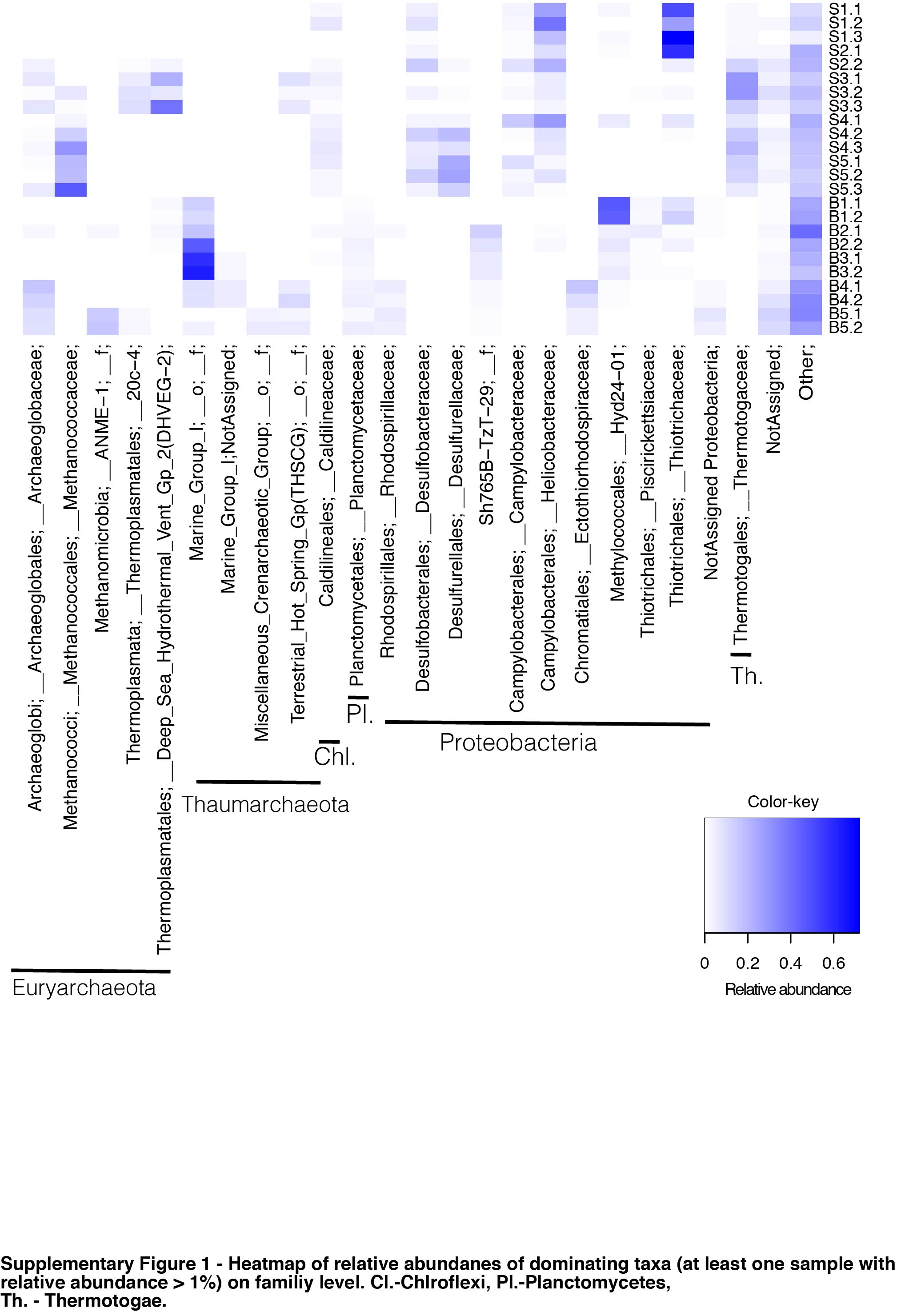
**

**
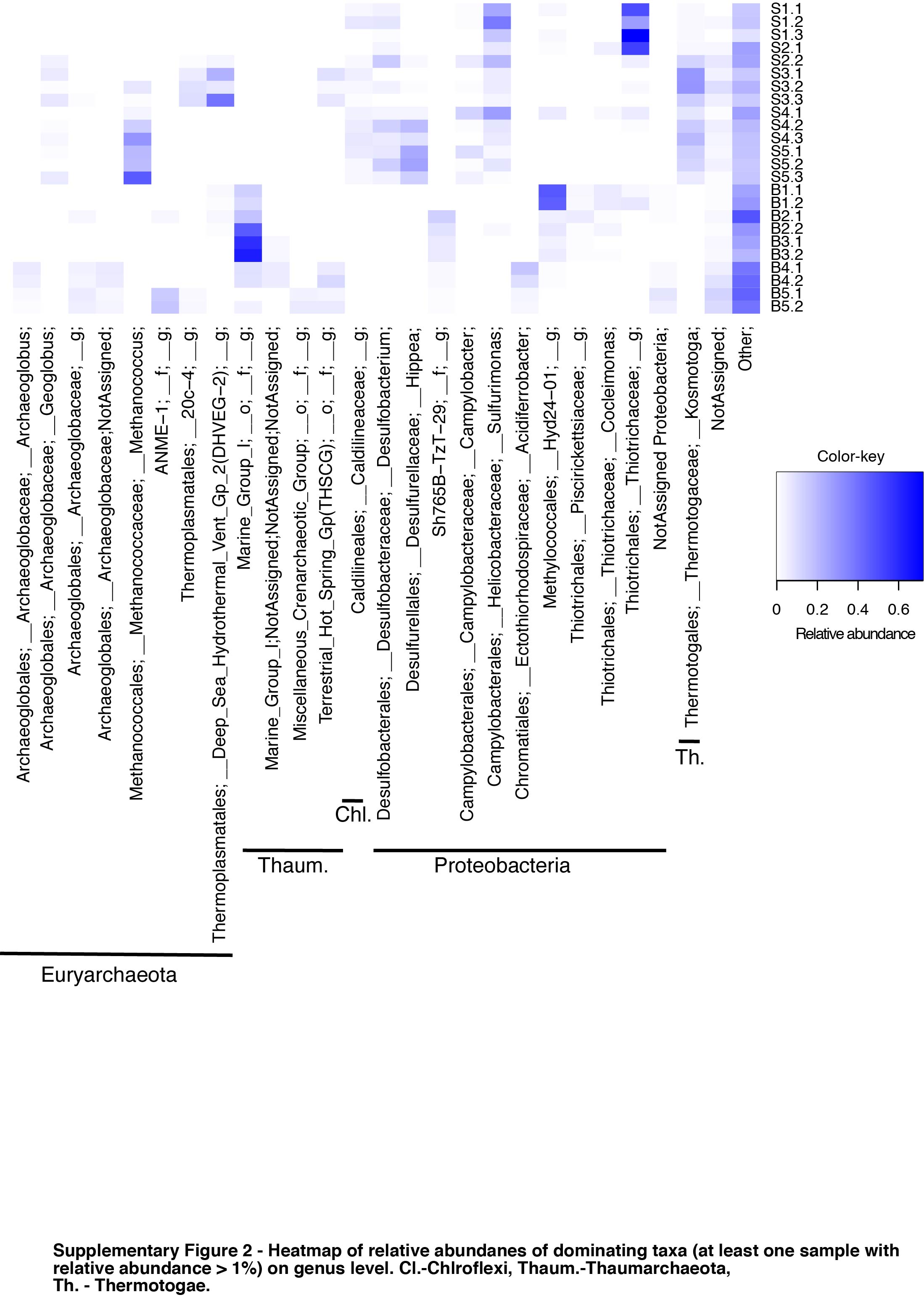
**
